# Multi-Mounted X-Ray Computed Tomography

**DOI:** 10.1371/journal.pone.0153406

**Published:** 2016-04-13

**Authors:** Jian Fu, Zhenzhong Liu, Jingzheng Wang

**Affiliations:** Research center of digital radiation imaging, Beijing University of Aeronautics and Astronautics, Beijing, People’s Republic of China; Chongqing University, CHINA

## Abstract

Most existing X-ray computed tomography (CT) techniques work in single-mounted mode and need to scan the inspected objects one by one. It is time-consuming and not acceptable for the inspection in a large scale. In this paper, we report a multi-mounted CT method and its first engineering implementation. It consists of a multi-mounted scanning geometry and the corresponding algebraic iterative reconstruction algorithm. This approach permits the CT rotation scanning of multiple objects simultaneously without the increase of penetration thickness and the signal crosstalk. Compared with the conventional single-mounted methods, it has the potential to improve the imaging efficiency and suppress the artifacts from the beam hardening and the scatter. This work comprises a numerical study of the method and its experimental verification using a dataset measured with a developed multi-mounted X-ray CT prototype system. We believe that this technique is of particular interest for pushing the engineering applications of X-ray CT.

## Introduction

X-ray computed tomography (CT) enables the non-destructive three-dimensional visualization of internal structures and is a powerful analysis tool [1–7]. It has been applied widely to clinic diagnosis [8–12], biomedical imaging [13–19], industry non-destructive testing [7, 20–22] and security inspection [23, 24] since its introduction in 1970s [25, 26]. CT has always fought against time to achieve improved scanning efficiency. The early development from the single pencil-beam translate/rotate 1st generation CT scanner to the electron-beam 5th generation scanner shows the efforts to reduce the scanning time. Each generation has been able to scan faster than its predecessor [27].

Over the last few decades, many novel CT techniques have been proposed to improve the imaging speed. Multi-slice CT (MSCT) [28, 29] has been successful in improving the scanning efficiency by a tremendous increase in the number of slices scanned simultaneously. Dual source CT [30] achieves a larger temporal resolution improvement. Single circular orbit cone-beam CT (CBCT) permits the production of hundreds of slices in parallel in a single gantry rotation and is regularly used to achieve high scanning efficiency [15, 20, 31–34]. Inverse geometry CT with stationary source array (SS-IGCT) [27, 35, 36] has been recently proposed to image a large volume of interest with minimal cone-beam artifacts and with very high temporal resolution. It frees the gantry to rotate very quickly and the rotation time is estimated to be as fast as 10 revolutions per second [27]. Although these techniques have improved tremendously the scanning efficiency, they work in single-mounted mode and need to scan the objects one by one. It may be reasonable for clinic diagnosis since the inspection task is specific for each patient. However, it is difficult to keep pace with the industrial mass production and not acceptable for the inspection in a large scale. Until now the conventional single-mounted CT (SMCT) is still applied to industry as a casual inspection technique not as a general tool.

In this paper, a multi-mounted CT (MMCT) method and its first engineering implementation are reported. The use of multi-mounted scanning geometry is the most iconic design and significantly different from the current CT systems. The rotation scanning of multiple objects can be executed simultaneously without the increase of penetration thickness and the signal crosstalk. The CT slice images of the inspected objects are reconstructed by the corresponding algebraic iterative reconstruction algorithm. Compared with the conventional single-mounted methods, it has the potential to improve the imaging efficiency and suppress the artifacts from the beam hardening and the scatter. This work comprises a numerical study of the method and its experimental verification using a dataset measured with a developed multi-mounted X-CT prototype system. This approach is of particular interest for pushing the engineering applications of X-ray CT. Although the rotation scanning of multiple objects can be also implemented at the same time by the conventional single-mounted CT systems when they are bundled together, it will lead to the increase of penetration thickness and the signal crosstalk. Furthermore, the beam hardening and the scatter will become much more complicated and the caused artifacts will harm the CT slices.

In the following sections, the multi-mounted scanning geometry and the corresponding algebraic iterative reconstruction algorithm are first described. Next by computer simulation, the validity of the proposed method is investigated. Finally the developed experimental system is described and the experimental results are presented. In this paper, our work focuses on the fan-beam case. It can be extended to the cone-beam case when area detectors are used to acquire the cone-beam projections and cone-beam algorithms to reconstruct slice images.

## Materials and Methods

### Scanning geometry

**Fig 1:**
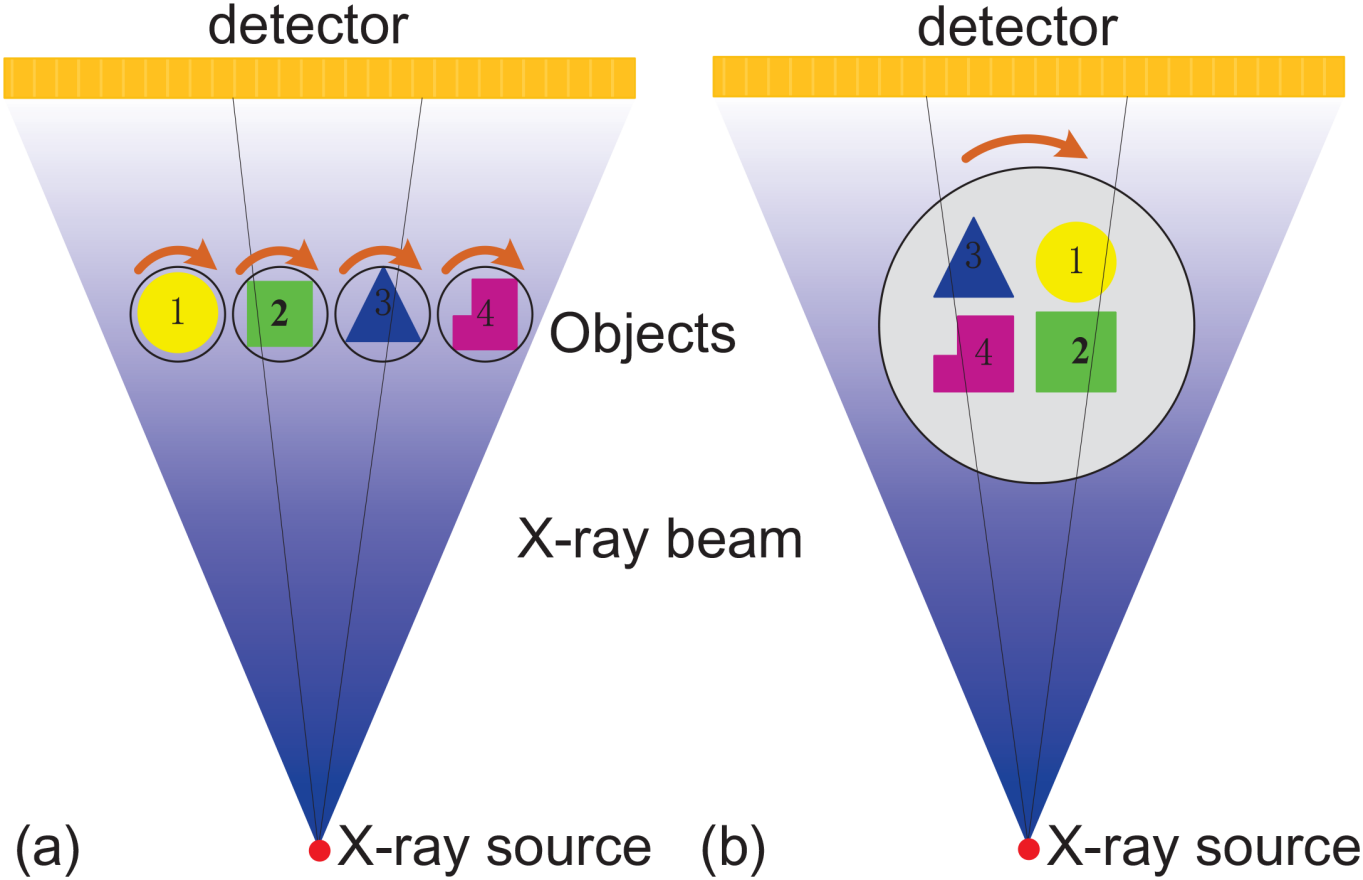
The two-dimensional fan-beam CT schematics. (a) is for the proposed multi-mounted CT and (b) for conventional single-mounted CT.

The two-dimensional fan-beam schematics for the proposed MMCT in comparison to conventional 3rd generation SMCT are illustrated in Fig 1. MMCT has multiple rotation tables along the direction parallel to the detector and can implement the rotation scanning of multiple objects. These tables can rotate simultaneously supported by the multi-axes numerical control synchronization technique. During the scanning, X-ray beam emitted from the X-ray source hits the inspected objects. The opposite detector records the attenuated X-ray beam by the objects and produces the projection dataset. Finally the CT slice images are reconstructed out from the recorded projection dataset with the CT reconstruction algorithms.

In conventional CT, there is only one rotation scanning table. So usually it only provides the inspection of one object one time. It means that the placement and scanning operations will be frequently repeated for the inspection in a large scale and limits the imaging efficiency. Although sometimes multiple objects can be scanned simultaneously when they are mounted together, depicted in Fig 1(b), it will increase the penetration thickness and lead to the signal crosstalk among these objects. The artifacts from the caused beam hardening and scatter will degrade severely the slice images. In contrast, MMCT permits the mount of multiple objects one time and also avoids the overlap among these objects. Consequently, the imaging efficiency is improved, the artifacts is suppressed and the signal crosstalk disappears.

The use of multi-mounted scanning geometry in MMCT is the most iconic design and significantly different from the current CT systems. It can provide dataset equivalent to that of a conventional SMCT scanner with multiple rotations, but it does so with a single multi-axes synchronous rotation. So it allows for a fast CT scanning and a reduction of artifacts caused by the increase of penetration thickness and the signal crosstalk.

### Reconstruction algorithm

There have mainly existed three kinds of CT reconstruction algorithms. The first kind is analytical methods such as filtered back-projection algorithms and back-projection filtered algorithms. Generally it works well when the projection dataset is complete and there is no mechanical error. The second one is based on algebraic iterative reconstruction such as algebraic reconstruction technique (ART), simultaneous ART and simultaneous iterative reconstruction technique (SIRT). This kind of methods can achieve better results than the analytical algorithms when applied to incomplete dataset. The last group is statistical iterative reconstruction. It performs well on suppressing noise.

The analytical methods requires that the central X-ray beam connecting the focus and the rotation center should be vertical to the detector. Obviously, this condition can not be satisfied in the proposed MMCT geometry depicted in Fig 1(a). The last group is usually involved with energy spectrum calculation to reconstruct high-quality images and the calculation burden is heavy. So ART algorithm, expressed in Eq (1), is adopted to reconstruct the slice images for the proposed MMCT.

$$\begin{array}{c} \left\{\begin{array}{c} f^{\left(0\right)},\;if\;k=0\\ f^{\left(k+1\right)}=f^{\left(k\right)}+r^{\left(k\right)}\frac{M_{k}}{\left|\right|M_{k}{\left|\right|}^{2}}\left(p_{k}-P_{k}\left(f^{\left(k\right)}\right)\right),\;otherwise. \end{array}\right. \end{array}$$ (1)

In Eq (1), *k* is a non-negative integer and represents the iteration reconstruction number. *f*^(0)^ is an initial image and *f*^(*k*)^ the *k*_*th*_ reconstructed image. *r*^(*k*)^ represents the *k*_*th*_ convergence coefficient ranging from 0 to 1. *M*_*k*_ is the projection matrix for the k_*th*_ ray and describes the contribution of all the image pixels to the k_*th*_ ray. *p*_*k*_ represents the measured projection data of the *k*_*th*_ ray and *P*_*k*_ the forward projection operator.

The whole reconstruction procedure of the MMCT technique involves two iterations: outer iteration and inner iteration. Eq (1) expresses the inner iteration. The outer iteration just repeats the calculation in Eq (1) if the iteration stopping criteria is not met after the last inner iteration. Obviously each inner iteration only uses the measured data of one ray to update the image. So this algorithm works in a ray by ray mode. So, in Eq (1), *k* represents the inner iteration number and equals the number of the rays which is the product of the number of detector pixels and the number of view angles. After the data of all the rays is handled, a complete inner iteration is realized. Next the whole procedure is repeated for another complete iteration and so on, until the convergence criteria is reached. For each inner iteration, this algorithm firstly calculates the forward projection of the current image and then compares this simulated projection to the corresponding measured data to get the correction value. Finally this correction value is added to the current image to obtain the updated image.

**Fig 2:**
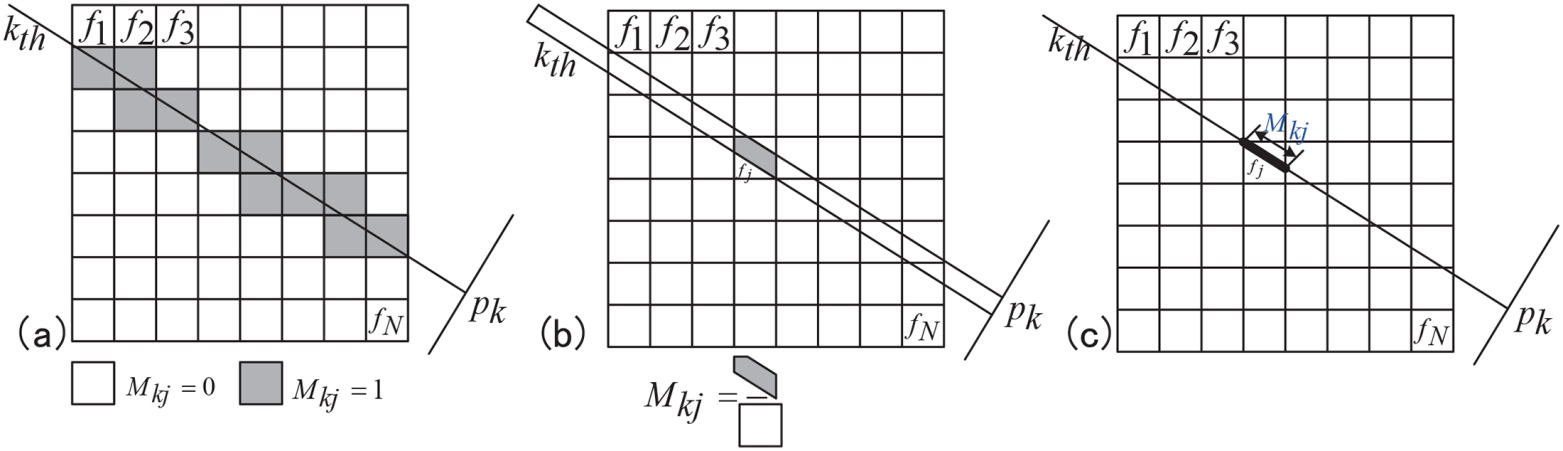
Three methods to obtain the projection matrix for a specific ray. (a) is based on passing or not, (b) area and (c) length.

For the k_*th*_ ray, the projection matrix *M*_*k*_ is actually an one-dimensional weighting factor matrix. Its size equals the number of the image pixels. There exist three methods to obtain this matrix, depicted in Fig 2. The one in Fig 2(c) is length-based and applied to the following simulations and experiments since it keeps a good balance between calculation efficiency and accuracy. The forward projection operation *P*_*k*_ represents the matrix calculation between the projection matrix *M*_*k*_ and the image matrix *f*. It can be expressed using Eq (2). In this equation, *N* is the number of the image pixels. *M*_*kj*_ is the weighting factor describing the contribution of the *j*_*th*_ image pixel *f*_*j*_ to the k_*th*_ ray.

$$\begin{array}{c} P_{k}=M_{k}\cdot f=\sum_{j=0}^{N}M_{kj}\times f_{j} \end{array}$$ (2)

The whole reconstruction procedure of the MMCT technique involves two iterations: outer iteration and inner iteration. Eq (1) expresses the inner iteration. The outer iteration just repeats the calculation in Eq (1) if the iteration stopping criteria is not met after the last inner iteration. So, in Eq (1), *k* represents the inner iteration number and equals the number of the rays which is the product of the number of detector pixels and the number of view angles. After the data of all the rays is handled, a complete inner iteration is realized. Next the whole procedure is repeated for another complete iteration and so on, until the convergence criteria is reached.

The standard derivation values of the three image matrixes of three neighboring outer iterations are calculated to monitor the convergence of the iteration reconstruction. It stops when the standard derivation value of the middle iteration is less than those of other two.

**Fig 3:**
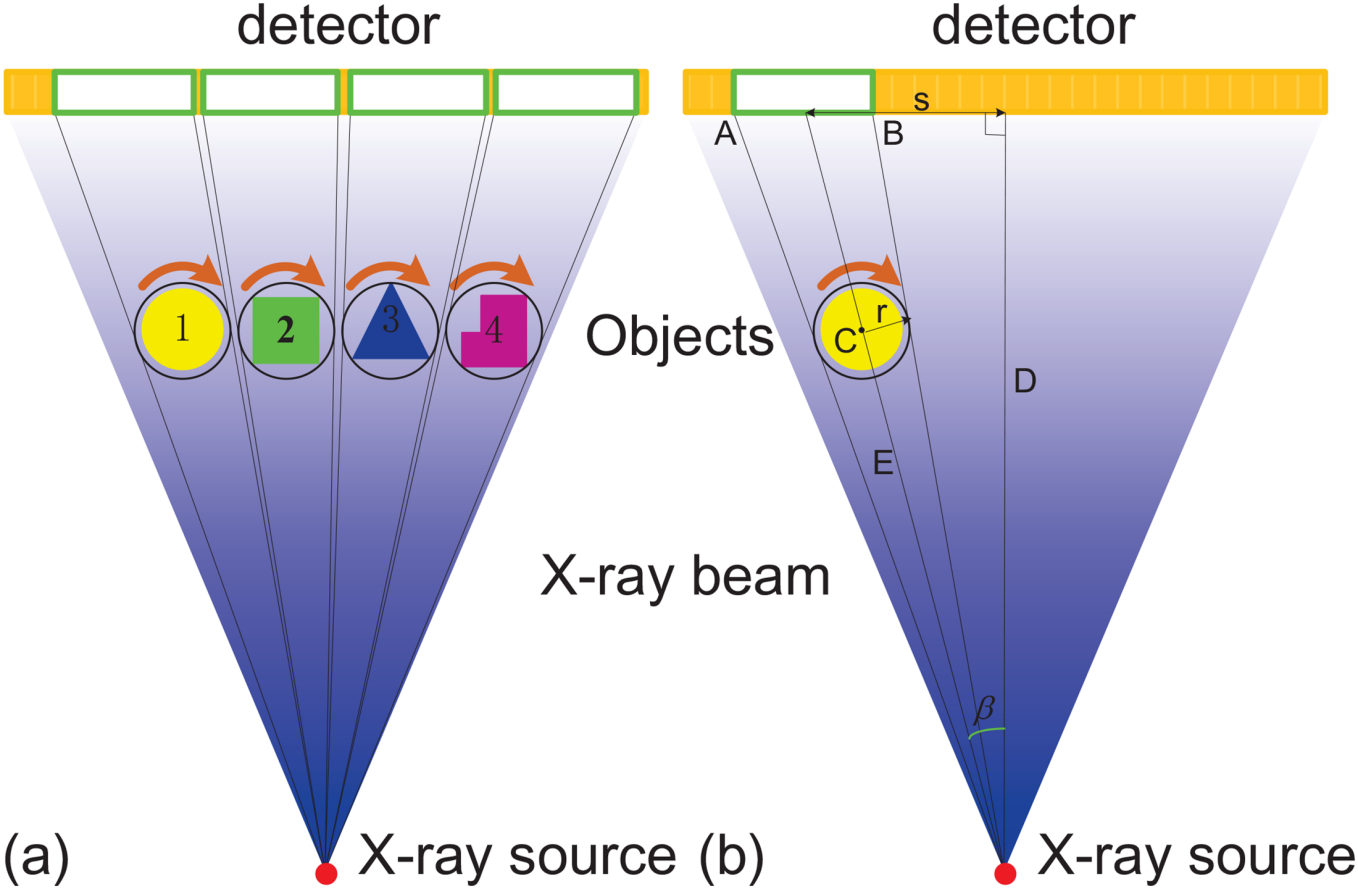
The schematics for projection split. (a) describes the segmented detector corresponding to different objects. (b) presents the determination of the detector segment for object 1. A and B are the end points of the detector segment for object 1, C is the rotation center, D the vertical distance from X-ray source to the detector, E the distance from X-ray source to the rotation center and r the rotation radius. s represents the distance from the detector center to the projection of the rotation center C. *β* represents the angle between the line connecting the focus to the rotation center and the line vertical to the detector passing through the focus.

For the proposed MMCT system in Fig 1(a), there are multiple rotation axes. Eq (1) can not be directly applied to the recorded MMCT projection dataset. It should be firstly split into multiple groups, depicted in Fig 3(a). Each one corresponds to one object. Then ART in Eq (1) is applied to these groups to reconstruct the slice images respectively. As an example, Fig 3(b) presents how to determine the group for object 1. The idea is to calculate the positions, *S*_*A*_ and *S*_*B*_, of the end points, A and B, of the the corresponding detector segment with given geometrical parameters *D*, *E*, *r* and *s*. This detector segment $\bar{AB}$ provides projections for object 1. The calculation formulas are expressed in Eqs (3) and (4). In practice, the values of *D*, *E* and *s* are measured with the well-known geometrical estimation methods [37, 38] and the value of *r* is set manually to avoid the overlap with neighboring detector segments.

$$\begin{array}{c} S_{A}=D\times tan\left(atan\frac{s}{D}+asin\frac{r}{E}\right) \end{array}$$ (3)

$$\begin{array}{c} S_{B}=D\times tan\left(atan\frac{s}{D}-asin\frac{r}{E}\right) \end{array}$$ (4)

Schematically, the whole reconstruction procedure of the MMCT technique is described as following:

(i) split the recorded projection dataset *p* into multiple groups with Eqs (3) and (4);
(ii) for each group, repeat the following step (iii)-(viii).
  (iii) assume an initial image *f*^(0)^ and set the iteration number *k* = 0;
  (iv) execute the operation *P*_*k*_(*f*^(*k*)^) to get forward projection $p_{k}^{\prime }$;
  (v) calculate the difference between the measured projection data *p*_*k*_ and $p_{k}^{\prime }$;
  (vi) modify the image by adding the difference to the pixels that contribute to the *k*_*th*_ ray;
  (vii) set *k* = *k*+1 and repeat steps (iv)-(vi) until all the rays are handled and a complete iteration is completed;
  (viii) set the current result as the initial image of next iteration to repeat steps (iii)-(vii) until the iteration reaches convergence.

## Numerical study

In this section, numerical simulations have been carried out to demonstrate the validity and the advantages of the proposed method. We restrict ourself to the case with four rotation axes. Four well-known two-dimensional Shepp-Logan phantoms [2] are scanned simultaneously. This phantom consists of ten ellipses with different diameters and grey values ranging from 0 to 1. The size of the phantom is set to 184×184 pixels, the number of detector channels to 1024 and the size of one channel to one pixel. The distance from source to detector is 4000 pixels and the rotation radius 128 pixels. The distances from the center of the detector to the projection position of the rotation centers are -384, -128, 128 and 384 respectively. The scanning angular range is [0° 359°] and the step angle is 1°. Firstly the line integral projection *p* is calculated analytically based on the exact mathematical descriptions of the phantom and the X-ray path. Then the projection dataset is split into four groups corresponding to four phantoms with Eqs (3) and (4). Next CT reconstructions with ART in Eq (1) is carried out and analyzed.

**Fig 4:**
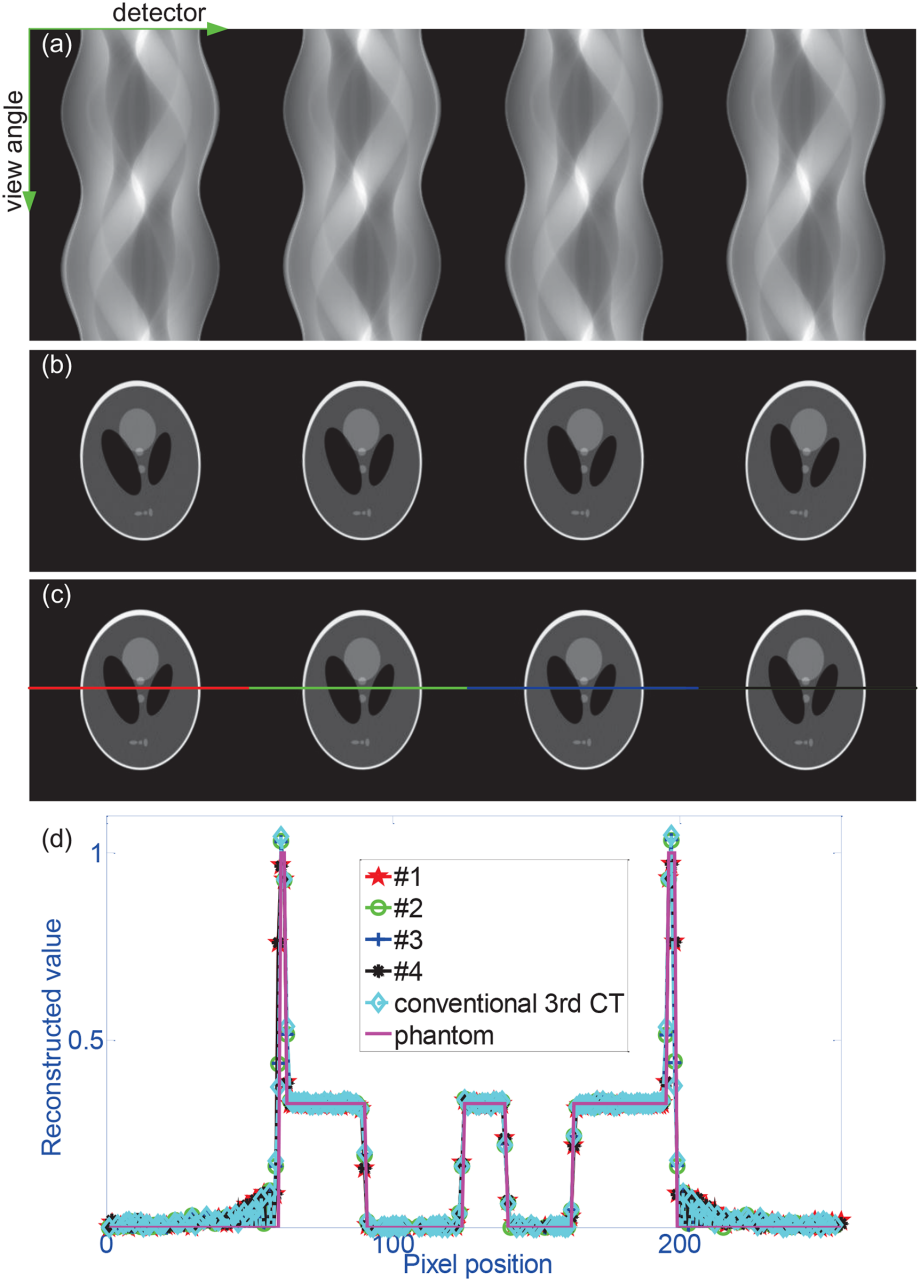
The simulation results. (a) shows the projection sinogram for four phantoms recorded by the detector in the MMCT system. (b) presents the reconstructed slice images of four phantoms. (c) displays the slice images with the correction of the initial scanning angular difference among these four phantoms. (d) presents the line profiles of the 128th row in these slice images with the phantom and the result from the conventional 3rd CT system. In (a), (b) and (c), the 1st, 2nd, 3rd and 4th columns correspond to object 1, 2, 3 and 4 respectively. The solid colored lines in (d) indicate the 128th row of the images.

Fig 4 displays the simulation results. Fig 4(a) shows the MMCT projection sinogram. Fig 4(b) presents the reconstructed images with ART in Eq (1). Clearly these slice images exhibit almost no disparity with the phantom except that each one has a different orientation. It is caused by the angle *β*, depicted in Fig 3(b), between the line connecting the focus to the rotation center and the line vertical to the detector passing through the focus. The value of this angle equals *atan*(*s*/*D*) and is different for each object. This problem can be resolved by setting the value of this angle to be the initial reconstruction angular position instead of zero. Fig 4(c) presents the corrected slice images. Fig 4(d) compares line profiles of the 128th row in these slice images with the original phantom and the result from the conventional 3rd CT system. We can find that they match the phantom well. Clearly the proposed MMCT methods yield acceptable reconstruction of the phantom with higher efficiency.

A quantitative comparison of the reconstruction results presented in Fig 4 is performed by calculating the *normalised root mean squared error (NRMSE)* [1], which is listed in Table 1. NRMSE is a bias measure of the reconstruction as compared to the phantom and is used to quantify the reconstruction error [1]. From Table 1, it is clear that the proposed method works well and has a performance similar to that of the conventional 3rd CT.

**Table 1:** The NRMSE values of the reconstructions in Fig 3.

Phantom	NRMSE
1	0.2965
2	0.2930
3	0.2939
4	0.2970
the one in conventional 3rd CT	0.3047

## Experiments

**Fig 5:**
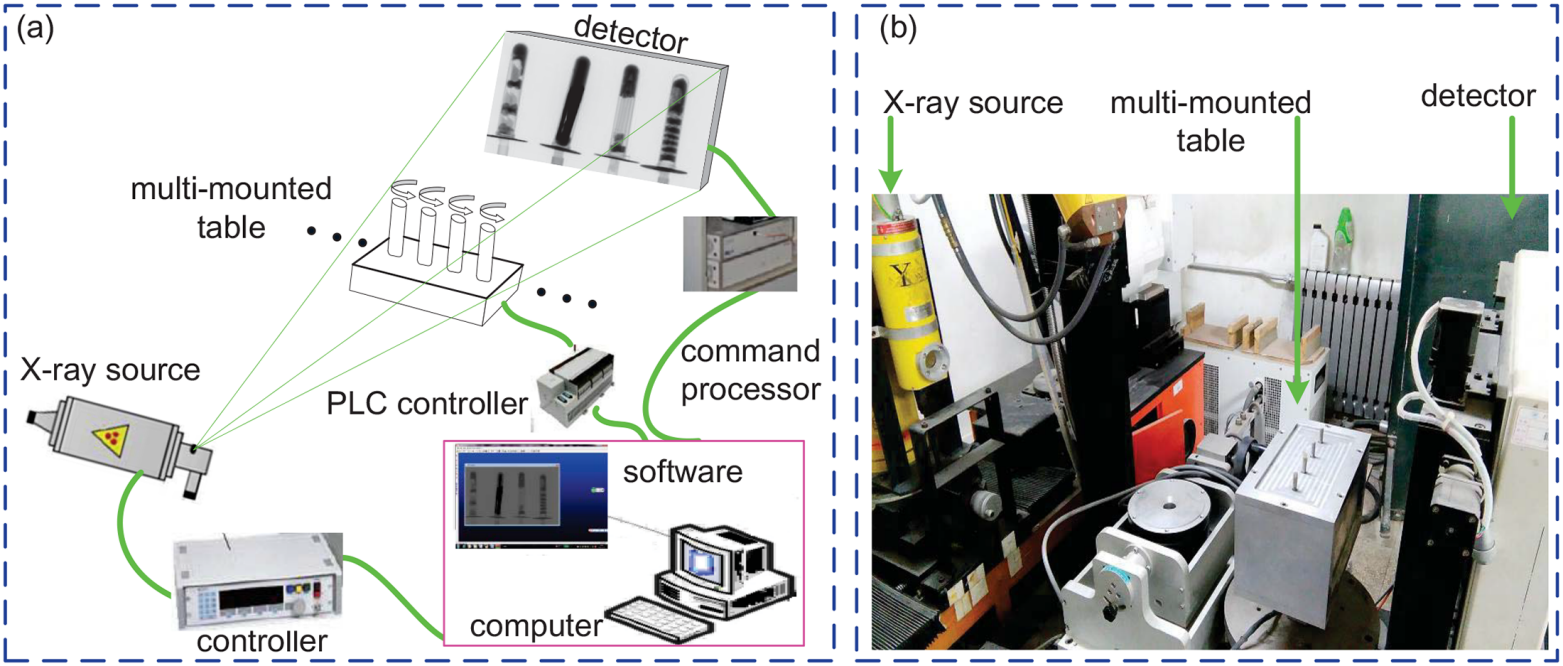
The developed MMCT system. (a) is the structural diagram of the developed MMCT system and (b) the photo.

We experimentally verified our conclusions drawn from the numerical studies by reconstructing the images from measured MMCT projection data of multiple samples. The experimental data used for the reconstruction was recorded at a MMCT system developed by us. It consists of an 160 keV X-ray source, YTU160-D01, from YXLON company (Germany), a flat panel detector (FPD), PaxScan2520, from VARIAN company (USA), a multiple-mounted rotation table and a computer. The X-ray source is a continuous one and the maximum power is 0.32 kW. The FPD has two working modes: internal trigger mode and external trigger mode. Considering the X-ray source is continuous, we select the internal trigger mode. The size of one detection element is 0.127 mm, the length of the detector 250 mm and the height 200 mm. The central row of the FPD is selected as the detector in the discussed two-dimensional fan-beam MMCT system and has 1920 detection elements. The multi-mounted table holds four samples and executes the rotation scanning simultaneously. The computer runs the system software to implement the mechanical movement control, the projection acquisition and the image reconstruction. Fig 5 shows the developed MMCT system.

**Fig 6:**
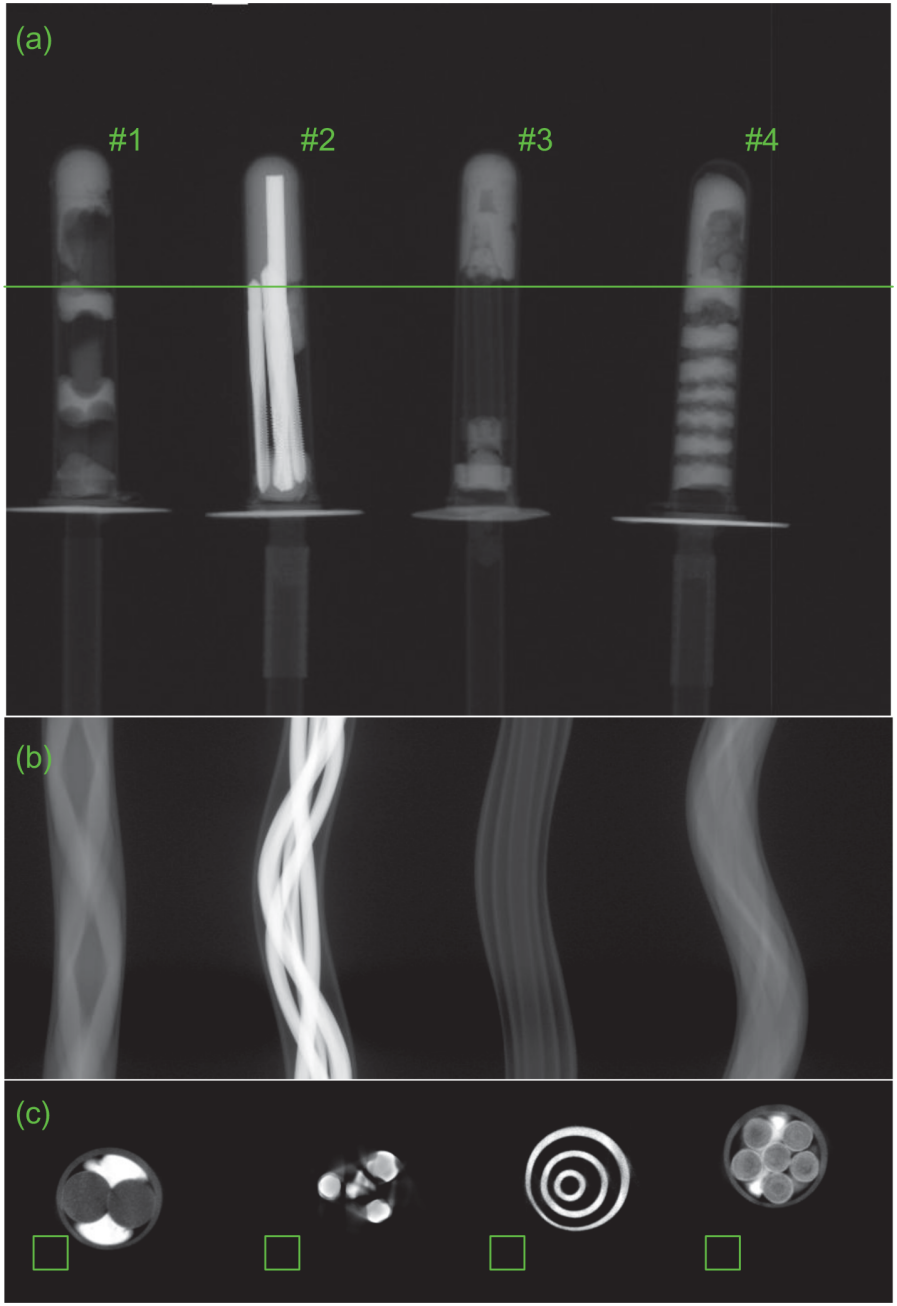
The multi-mounted CT experimental results. (a) is one of the two-dimensional projection images, (b) the projection sinogram and (c) the reconstructed images with ART in Eq (1).

In the experiment, four plastic tubes, full of different material structures respectively, were mounted in the tables. Indicated in the projection image Fig 6(a), the 1st one contains capsules and plasticine, the 2nd steel drilling bits and plasticine, the 3rd a ball-pen body and plasticine and the fourth pills and plasticine. These specimens ensure that the advantages of the proposed methods are visualized properly. The distance from source to detector was 1250mm and from detector to rotation centers 210mm. The angular increment was 0.5° and 720 projections was recorded over 360°. The system operated at 115kV with a tube current of 1.8mA.

Fig 6 displays the MMCT experimental results. Fig 6(a) is one of the two-dimensional projection images recorded by the FPD. Fig 6(b) shows the MMCT projection sinogram. Fig 6(c) presents the reconstructed images with ART in Eq (1). Obviously the cross-sections of the 1st, 3rd and 4th tubes are reconstructed accurately and the structure details can be clearly recognized. The image of the 2nd tube has visible artifacts and the structure of the tube can not be observed. It is caused by the steel drilling bits and the followed beam-hardening and scatter. However, it doesn’t affect the neighboring tubes. So there is no signal crosstalk between this tube and other ones.

**Fig 7:**
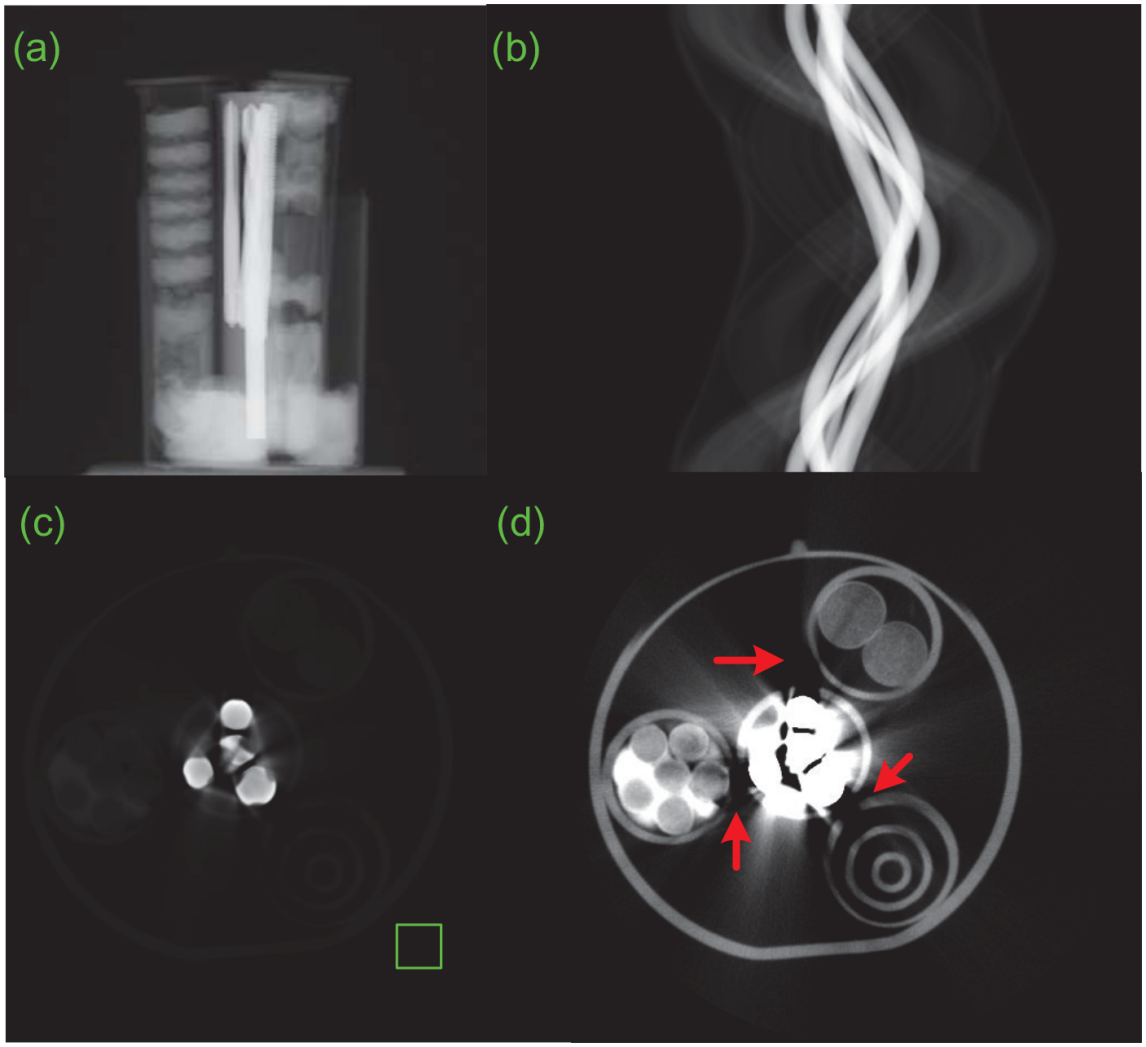
The conventional 3rd CT experimental results. (a) is one of the two-dimensional projection images, (b) the projection sinogram and (c) the reconstructed images with ART in Eq (1). (c) is displayed into (d) by histogram equalization.

To make a comparison, these four tubes were bundled together in a bigger tube and scanned. Fig 7 displays the experimental results. Fig 7(a) is one of the two-dimensional projection images recorded by the FPD. Fig 7(b) shows the projection sinogram. Fig 7(c) presents the reconstructed images with ART in Eq (1). Fig 7(c) is displayed into Fig 7(d) by histogram equalization to show more structures. As indicated by the red arrows in Fig 7(d), the artifacts from the steel drilling bits rush into regions of other three tubes and wipe away some structures. Obviously the increase of the penetration thickness and the signal crosstalk happened. The former deteriorates the beam-hardening and scatter and leads to more artifacts. The latter provides these artifacts a chance to affect neighboring structures. In contrast, these problems are avoided in the proposed MMCT system. These experimental results in Figs 6 and 7 indicate that, compared with the conventional single-mounted CT, the multi-mounted CT technique has the potential to improve the imaging efficiency and suppress the artifacts from the beam hardening and the scatter.

Table 2 lists the standard derivation values of background regions marked by the rectangles in Figs 6 and 7. The values corresponding to objects 1, 3 and 4 in Fig 6 are much less than the one in Fig 7. It shows that the proposed method has less artifacts and performs better than the conventional method.

**Table 2:** The standard derivation values of background regions marked by the rectangles in Figs 6 and 7.

regions	standard derivation values
1 in Fig 6	6.2645e-8
2 in Fig 6	1.0828e-5
3 in Fig 6	2.2853e-8
4 in Fig 6	6.7379e-8
the one in Fig 7	4.5373e-4

## Discussion and Conclusion

In summary, we have proposed a multi-mounted CT technique and demonstrated its validity both numerically and with real experimental data. This method adopts a multi-mounted scanning geometry to acquire the projections of multiple objects simultaneously without the increase of penetration thickness and the signal crosstalk. It is the most iconic design and significantly different from the current CT systems. The use of the multi-mounted scanning geometry provides the MMCT system the potential to improve the imaging efficiency and suppress the artifacts from the beam hardening and the scatter. We envisage that, particularly for industrial mass production, where CT has been proven to be a uniquely powerful technique [16], this approach will provide a possible solution for the CT inspection in a large scale and push X-ray CT towards future applications as a general tool.

In the principle verification experiment of our method, four objects were inspected simultaneously. Actually it permits the inspection of much more objects with elaborative layout of the multi-mounted scanning geometry. Ignoring the geometrical magnification, Eq (5) gives out how to calculate the maximum number of objects inspected simultaneously in the MMCT system. The derivation of this equation is presented in the appendix. In this formula, *int*[] represents the rounding operation, L is the length of the detector, r the rotation radius and D the vertical distance from X-ray source to the detector. Taking the CT inspection of nuclear fuel rod cladding tubes as an example [16], the number of inspected objects simultaneously can reach about 20 where the values of L, r and D are typically 200mm, 5mm and 1000mm respectively [16]. Obviously, the proposed MMCT system can achieve an imaging efficiency orders of magnitude greater than the conventional methods and shows a promising application future. When cone-beam CT algorithms are applied to the recorded projections, this technique can be extended from two-dimensional fan-beam to three-dimensional cone-beam.

$$\begin{array}{c} n=1+int[\left(L-\frac{2r\sqrt{D^{2}+L^{2}/4}}{D}\right)/\left(2r\right)] \end{array}$$ (5)

Eq (5) involves the geometrical layout parameters of the proposed MMCT system and can work also as an optimal design model. Parameter r is the rotation radius and actually also represents the specific maximum field of view of each object. Given the system geometrical layout and the maximum number of objects inspected simultaneously, the maximum field of view of each object r can also be calculated out. This equation will be useful in developing this kind of MMCT imaging system.

## Appendix

**Fig 8:**
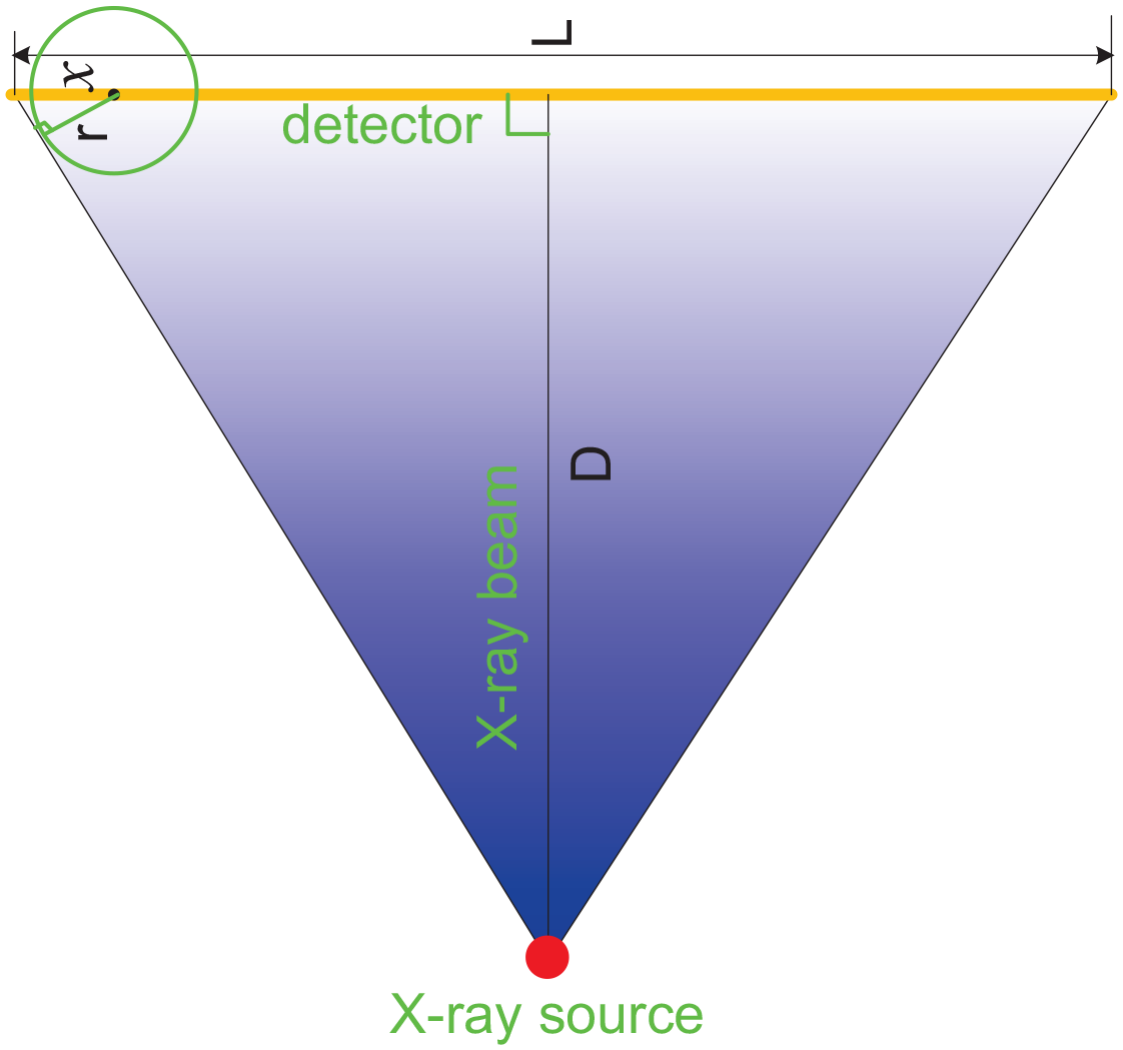
The figure used for calculating the maximum number of inspected objects simultaneously by the MMCT system. Here the rotation center is shifted to detector and geometrical magnification is ignored.

According to Fig 8, we have

$$\begin{array}{c} n=1+int[\frac{L-2x}{2r}] \end{array}$$ (6)

and

$$\begin{array}{c} \frac{r}{D}=\frac{x}{\sqrt{D^{2}+L^{2}/4}}. \end{array}$$ (7)

Here *int*[] represents the rounding operation and *n* is the maximum number of objects inspected simultaneously by the MMCT system. From Eq (7), we obtain

$$\begin{array}{c} x=\frac{r\sqrt{D^{2}+L^{2}/4}}{D}. \end{array}$$ (8)

Finally, we get Eq (5) by replacing *x* in Eq (6) with Eq (8)
